# Perfluorooctanesulfonic Acid Detection Using Molecularly Imprinted Polyaniline on a Paper Substrate

**DOI:** 10.3390/s20247301

**Published:** 2020-12-19

**Authors:** Ting-Yen Chi, Zheyuan Chen, Jun Kameoka

**Affiliations:** 1 Department of Materials Science and Engineering, Texas A&M University, College Station, TX 77843, USA; kevin0149@tamu.edu; 2 Department of Electrical and Computer Engineering, Texas A&M University, College Station, TX 77843, USA; zychen@tamu.edu

**Keywords:** molecularly imprinted polymer, polyaniline, perfluorooctanesulfonic acid, low-cost, paper sensor

## Abstract

Perfluorinated compounds like perfluorooctanesulfonic acid (PFOS) are synthetic water pollutants and have accumulated in environments for decades, causing a serious global health issue. Conventional assays rely on liquid chromatography and mass spectroscopy that are very expensive and complicated and thus limit the large-scale monitoring of PFOS in wastewater. To achieve low-cost and accurate detection of PFOS, we designed a paper-based sensor with molecularly imprinted polyaniline electrodes that have recognition sites specific to PFOS. The calibration curve of resistivity ratios as a function of PFOS concentrations has a linear range from 1 to 100 ppt with a coefficient of determination of 0.995. The estimated limit of detection is 1.02 ppt. We also investigated attenuated total reflectance Fourier-transform infrared spectroscopy (ATR-FTIR) and X-ray photoelectron spectroscopy (XPS) spectra of the surface of the polyaniline (PANI) electrodes to propose the potential recognition sites in polyaniline matrix and the detection mechanism. This electrical paper sensor with low cost and excellent sensitivity and selectivity provides the potential for large-scale monitoring of wastewater.

## 1. Introduction

Pollution in the environment has become a worldwide issue. Chemical pollutants in air [1,2] and heavy metals [3] as well as organic dyes [4,5,6] in water are of the greatest concern and responsible for a massive loss of lives. Among those pollutants are perfluorinated compounds (PFCs), a series of aliphatic chemicals in which fluorine atoms are substituted for all or some of the hydrogen atoms on the alkyl groups. PFCs have been used in industries for manufacturing water and oil foiling products, fire-retardant materials, and packaging since the 1950s [7,8]. After decades of utilization in large quantities, due to their extraordinary chemical and thermal stability, PFCs were found to be ubiquitously persistent in environments and cumulative in different organisms [9,10,11], resulting in toxicity to metabolism [12], reproduction [13], and liver function [14]. Among a series of PFCs with various chain lengths, the eight-carbon group is of the most interest and is responsible for the largest concentrations monitored in environments because of their more effective activities and larger production scale than any other homologues [15,16,17]. As a result, governmental regulations for maximum tolerance of PFCs, particularly of perfluorooctanesulfonic acid (PFOS), are being legislated and updated frequently. For example, the United States Environmental Protection Agency (USEPA) has released the advisory maximum concentration of 70 ng/L (70 part-per-trillion, 70 ppt) for a combination of perfluorooctanoic acid (PFOA) and PFOS in drinking water [18]. According to the guidance broadcast by the Interstate Technology and Regulatory Council, approximately six million people in the United States are consuming drinking water in which the combined concentration of PFOA and PFOS exceeds the health advisory of 70 ppt from the USEPA [19]. In addition, PFOS and its relatives have been investigated by and are included in the list of the Stockholm Convention on Persistent Organic Pollutants, which calls for eliminating the production of and establishing regulatory standards for certain pollutants [15,20]. Therefore, due to the severe public health issues around the world, the detection of PFCs in water is in urgent demand. Currently, the detection of PFCs mostly depends on high performance liquid chromatography/mass spectroscopy (HPLC/MS) [21,22] and gas chromatography/mass spectroscopy (GC/MS) [23,24] which are very expensive and time-consuming. As a result, it is of interest to develop an economical assay with the capability of detecting notorious chemicals without sacrificing sensitivity and reliability. Recently, several sensors utilizing the optical changes of quantum dots and chromophores to detect PFCs have been reported [25,26,27]. However, these optical indicators could be vulnerable in harsh environments and their durability is a concern. Additional steps and accessories for sample preparation and detection are needed, making the sensors less applicable for widespread use. Consequently, an accurate, reliable, and fast assay for detecting PFCs is still in thriving development.

Molecular imprinting technology as a promising method for creating binding sites specifically for templates has emerged in the last decades. Its history can be tracked back to the 1940s when Linus Pauling introduced the concept of molecularly imprinted polymers (MIPs) for synthesizing artificial antibodies for molecular templates [28,29]. Based on the general principle of molecular imprinting, monomers are polymerized in the presence of templates, followed by the removal of templates, leaving cavities as complements to target analytes [30,31]. For electrochemical sensors using MIPs, conductive polymers are desirable because of their capability in electron-conducting through the bonding sites and matrix, leading the signal variation upon the recognition of target molecules [32,33,34,35]. Polyaniline (PANI), one of the typical conductive polymers, gives a promising potential for being introduced by a molecular imprinting process because of economic and simple synthesis/doping procedures [32,36,37,38]. The excellent capacitive nature and rapid charge/discharge capability of electroactive PANI provides applications such as supercapacitors [39,40,41,42] and controlled drug release systems [43]. In addition, the excellent environmental durability and availability for operating in aqueous phase make PANI one of the best candidates for molecularly imprinted conductive polymers [44]. Various research of molecularly imprinted PANI (MIP-PANI) sensors for detecting chemicals [45,46,47,48] and proteins [37,49] has been reported. However, the synthesis of MIP-PANI usually relies on electrochemical polymerization, in which the substrates must be conductive materials like precious metals or graphite, potentially increasing the cost of the entire sensor.

Paper is flexible, portable, low-cost, lightweight, biodegradable, and easily processed [50]. In particular, the low cost enables paper sensors to compete with conventional assays that are expensive and have a high threshold. For example, a single HPLC/MS assay could range from tens to hundreds of dollars; however, paper-based sensors are expected to cost much less than one dollar per device [51,52,53,54]. Especially for those underdeveloped countries and areas, the high demand for a simple and sensitive device at an affordable price is of utmost interest. Moreover, paper-based devices can be easily fabricated by inkjet printing or by screen printing conductive materials on any paper substrates [55]. Conductive inks and printers are commercially available for printing electrodes with a variety of shapes as needed, giving engineers exceptional flexibility in designing paper-based electrodes without sacrificing conductivity [56]. Together with these advantages, various paper-based sensors are reported as a potential platform for replacing conventional substrates such as printed circuit boards or silicon or glass substrates [57,58,59,60,61].

In this study, we utilized molecular imprinting technology to synthesize PFOS molecularly imprinted polyaniline (PFOS-MIP-PANI) and designed a paper-based sensor incorporated with PFOS-MIP-PANI electrodes for the detection of PFOS. PFOS-MIP-PANI was synthesized on paper strips in the presence of PFOS, followed by removing PFOS molecules from the polymer matrix. Resistances of PFOS-MIP-PANI as electronic responses upon the exposure to levels of concentration of PFOS were evaluated to acquire the calibration curve, minimum detection limit, and the linear regression range. Surface characterizations on the PFOS-MIP-PANI electrodes were also investigated to propose the possible detecting mechanism of PFOS with PANI polymer. The simple and cost-effective fabrication process allows the paper-based PFOS-MIP-PANI sensor to be capable of serving as a low-cost, accurate, and standard assay for PFOS detection and monitoring in aqueous samples.

## 2. Materials and Methods

### 2.1. Materials

Aniline, ammonium persulfate (APS), perfluorooctanesulfonic acid (PFOS), perfluorooctanoic acid (PFOA), perfluorobutanoic acid (PFBA), and perfluorohexanoic acid (PFHxA) were purchased from Sigma-Aldrich (St. Louis, MO, USA). Hydrochloric acid (HCl, 36–38%) and acetic acid were purchased from Macron (Center Valley, PA, USA). Methanol was obtained from VWR Chemicals (Radnor, PA, USA). Paper made of polyester fibers was obtained from Xerox (Norwalk, CT, USA). Silver conductive ink (Cat# 125-15) was purchased from Creative Materials (Ayer, MA, USA).

### 2.2. Synthesis of Molecularly Imprinted Polyaniline on Paper

**Figure 1:**
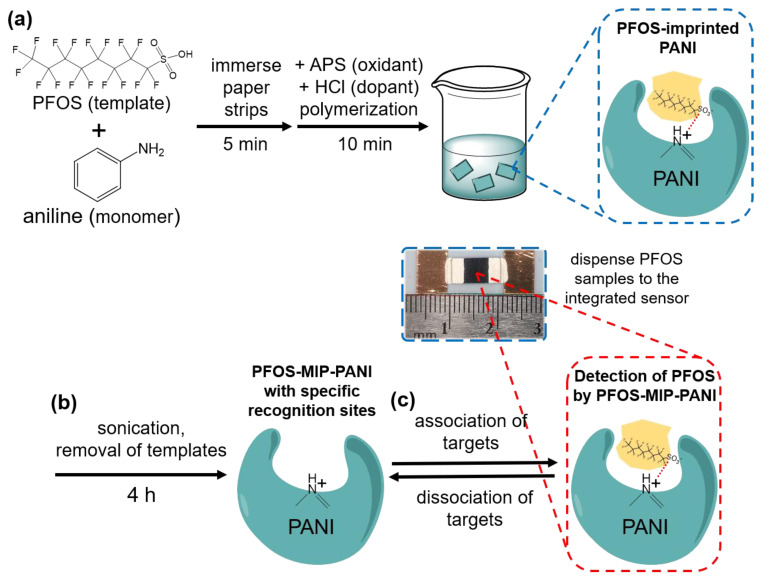
The schematic diagram of the synthesis of PFOS-MIP-PANI and the molecular imprinting process. (**a**) PFOS, as the template, was first mixed with aniline monomers in HCl aqueous solution, followed by immersing paper strips for the thorough absorption of the monomers on the surface. The polymerization was initiated by adding the oxidant, and the PFOS-imprinted polyaniline (PANI) was synthesized. (**b**) The PFOS templates were removed by sonication for 4 h in a mixed solution of acetic acid and methanol at a *v*/*v* ratio of 1:6, leaving cavities of specific recognition sites. (**c**) PFOS samples were then dispensed to the surface of the integrated paper sensor, and the PFOS-MIP-PANI was then able to detect PFOS by the molecularly imprinted structures. PFOS targets and PFOS-MIP-PANI performed an equilibrium system of association and dissociation.

PFOS was used as the template for the molecular imprinting process on the PANI. The monomer solution was prepared by mixing 0.2 g of aniline and 1 mL of 100 ppm PFOS aqueous solution in 1 M HCl with a final volume of 5 mL. Paper strips with the dimensions of 1 cm × 0.5 cm were immersed in the monomer solution and soaked for 5 min to be saturated with the solution. The polyester paper we used can absorb the most PANI on its surface during polymerization, giving the best conductivity and comparing to other filter papers such as the Whatman series. Afterwards, the oxidant solution prepared by mixing 409 mg of APS in 5 mL of 1 M HCl was added drop-by-drop into the monomer solution under vigorous stirring to initiate the polymerization of PANI on paper substrates. The HCl was to act as the dopant enriching the conductivity of the PANI and to sustain the pH (pH = 0) in the reaction. The concentration of PFOS templates in the reaction solution was 10 ppm. After 10 min of polymerization, the paper strips were removed and flushed with deionized water (DI water) multiple times until the eluent was clear and no excess PANI as dark-colored particles was found in the solution. To remove the template from the PANI matrix, the paper strips were immersed in a mixture of methanol and acetic acid at a ratio of 6:1 (*v*/*v*), followed by sonication for 4 h. The solution was refreshed every hour to ensure the effectiveness of template extraction. The resulting strips were removed and rinsed with DI water until the pH reached 7, and then air-dried at 25 °C for at least 12 h before fabrication. Non-molecularly imprinted PANI, serving as the control, was synthesized by following the same protocol given above without the addition of PFOS in the monomer solution. The schematic diagram of the synthesis of PFOS-MIP-PANI and the principle of molecular imprinting technology is shown in Figure 1.

### 2.3. PFOS Paper Sensor Fabrication

For fabricating the device, a PANI pad was first attached to a piece of stencil with the dimensions of 1 cm × 3 cm using double-sided tape. Two pieces of copper tape (1 cm × 1/4 in), as the connecting electrodes, were then attached on both sides of the PANI pad, leaving a 1 mm gap between the PANI pad and each copper electrode. The gaps were filled with silver ink to build the conjunction between the PANI pad and the electrodes. The silver ink was then air-dried at 25 °C for at least 12 h, and the assembled device was ready to detect PFOS. The photographic image of the integrated sensor is shown in Figure 1.

### 2.4. PFOS Exposure and Resistivity Measurement

PFOS solutions with multiple concentrations from 0 to 100 ppt were prepared to cover the maximum tolerance of 70 ppt from the USEPA’s guideline. For the exposure step, a 30-μL aliquot of PFOS was dispensed on the center of the PANI pad and reacted for 30 min, and then air-dried at 25 °C. Relevant PFCs of PFBA, PFHxA, and PFOA were used for the selectivity experiment in which the concentration was 70 ppt corresponding to the USEPA’s tolerance. The change in resistance between the two electrodes after exposure to PFOS was measured by the direct current (DC) resistance mode of a multimeter (8846A, Fluke, Everett, WA, USA). Resistance (R) can be converted to resistivity (ρ) by using the following equation,

ρ = RA/L(1)

where L is the length and A is the cross-sectional area of the PANI paper electrode. Since the dimension of paper electrodes remain the same for all specimens, the measured resistance is proportional to the resistivity of the PANI paper electrodes. All the responses were normalized based on the DI water (0 ppt) by using the following equation, where ρ_before_ and ρ_after_ are the resistivity of the PANI paper electrodes before and after the exposure, respectively:

ratio_sample_/ratio_water_ = (ρ_after, sample_/ρ_before, sample_)/(ρ_after, water_/ρ_before, water_) (2)

The statistical analysis and the calibration curves were generated by using the default linear regression model in SigmaPlot. The statistical significance between experimental groups was defined as *p* (*p*-value) < 0.05 and determined by a Student’s *t* test performed by SigmaPlot. To estimate the limit of detection (LoD), the limit of blank (LoB) is first determined by [62],

LoB = μ_b_ + σ_b_ (3)

where μ_b_ and σ_b_ are the mean value and the standard deviation of blank samples, respectively. The limit of detection is therefore defined as [62],

LoD = LoB + σ_s_ (4)

where σ_s_ is the standard deviation of the sample of a lower concentration. By plugging in the mean value and standard deviation from the calibration curve into Equations (3) and (4), the limit of detection of PFOS for the PFOS-MIP-PANI paper sensor can be estimated. Regarding the long-term stability experiment for validating the durability of the synthetic PANI paper strips made using the above protocol, the DC resistance of the PANI paper strips was measured weekly at an ambient air temperature and the experiment lasted for 11 weeks from synthesis.

### 2.5. Fourier-Transform Infrared Spectroscopy (FTIR) Analysis

The surface of the PFOS-MIP-PANI sensing element was characterized by an attenuated total reflectance module of an FTIR spectrometer (ATR-FTIR, Nicolet 380, Thermo Scientific, Waltham, MA, USA) to evaluate the potential binding sites for PFOS on PFOS-MIP-PANI. The sensing part on the PANI pad was exposed to 150 ppt PFOS aqueous solution for 30 min and air-dried at 25 °C for at least 12 h, followed by an additional vacuum drying process for 2 days to ensure that there was no moisture in the specimens. The FTIR spectra were based on 64 scans and were collected at 25 °C with wavelengths from 500 cm^−1^ to 4000 cm^−1^ and at a resolution of 2 cm^−1^.

### 2.6. X-ray Photoelectron Spectroscopy (XPS) Analysis

To evaluate the effectiveness of removing PFOS from the PANI matrix, the XPS spectra of the surface of PFOS-MIP-PANI at different fabrication steps (before sonication, after sonication, and after sample dispensing) in the molecular imprinting process were obtained by an XPS system (Scienta Omicron, Denver, CO, USA) equipped with Mg/Al X-ray source (DAR 400, Scienta Omicron, Denver, CO, USA). Prior to XPS measurement, the specimens from the three steps were vacuum-dried for 4 days to remove any trace level moisture in the specimens. The binding energies of interests in the XPS spectra come from 1 s orbitals of fluorine (F1s) and nitrogen atoms (N1s), which were selected in the corresponding modes in the control panel of the XPS system.

### 2.7. Scanning Electron Microscopy (SEM) Analysis

After the exposure to 150 ppt PFOS for 30 min, the PFOS-MIP-PANI strips were air-dried at 25 °C for 12 h, followed by further drying under vacuum for additional 4 days. The specimens were then mounted on aluminum stubs using carbon tape and pre-treated with Pt/Pd sputtering. The surface morphology of PFOS-MIP-PANI was analyzed by a filed-emission scanning electron microscope (LYRA3, TESCAN, Czech Republic) at 10 kV.

## 3. Results

### 3.1. ATR-FTIR Spectra

**Figure 2:**
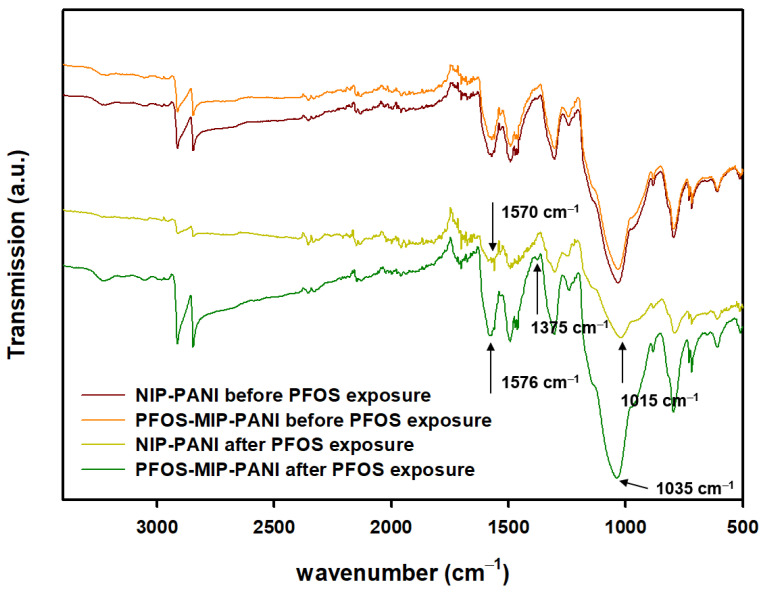
ATR-FTIR spectra of the surface of PFOS-MIP-PANI and NIP-PANI. NIP-PANI denotes the polyaniline synthesized in the absence of PFOS templates while the template removal process including sonication was still performed. The *y* axis based on the transmission mode has been adjusted in order to compare wavelengths of the characteristic peaks.

The ATR-FTIR spectra (Figure 2) of the surface of PANI electrodes after being exposed to PFOS show a characteristic peak around 1570 cm^−1^ and a red-shift from 1570 cm^−1^ to 1576 cm^−1^ while molecular imprinting process was applied to the PANI. In addition, the spectrum of PFOS-MIP-PFOS after PFOS exposure shows a characteristic peak at 1375 cm^−1^, which is not found in the spectrum of non-imprinted PANI (NIP-PANI). Another group of characteristic peaks at around 1015 cm^−1^ and 1035 cm^−1^ are also present in the spectra of PFOS-MIP-PANI and NIP-PANI.

### 3.2. XPS Spectra

**Figure 3:**
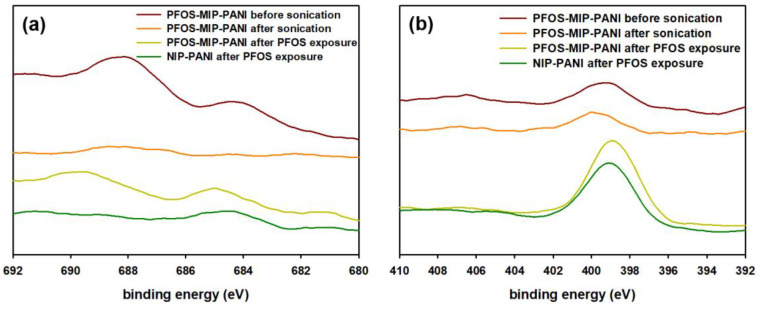
XPS spectra of the surface of PFOS-MIP-PANI and NIP-PANI. (**a**) F1s bonding. (**b**) N1s bonding.

The XPS spectra of the surface of PFOS-MIP-PANI and NIP-PANI are shown in Figure 3. Two sets of spectra regarding two types of binding energies from 1s orbitals of fluorine (F1s) and nitrogen (N1s) are presented. For PFOS-MIP-PANI before sonication, the spectrum of F1s bonding shows peaks at 688 eV and 684 eV; however, the peaks disappear after the PANI strips were treated by sonication and reappear after the exposure to PFOS aqueous solution. The NIP-PANI, acting as the control, shows no peak at 688 eV, and the peak at 684 eV is not as significant as that of PFOS-MIP-PANI. On the other hand, the N1s bonding spectra show peaks around 399 eV for all the conditions and types of PANI electrodes. After the exposure of PFOS, PFOS-MIP-PANI exhibits a red-shift of the peak at 399 eV when compared with the peaks of NIP-PANI and PFOS-MIP-PANI before the exposure of PFOS.

### 3.3. Scanning Electron Microscopy Images

**Figure 4:**
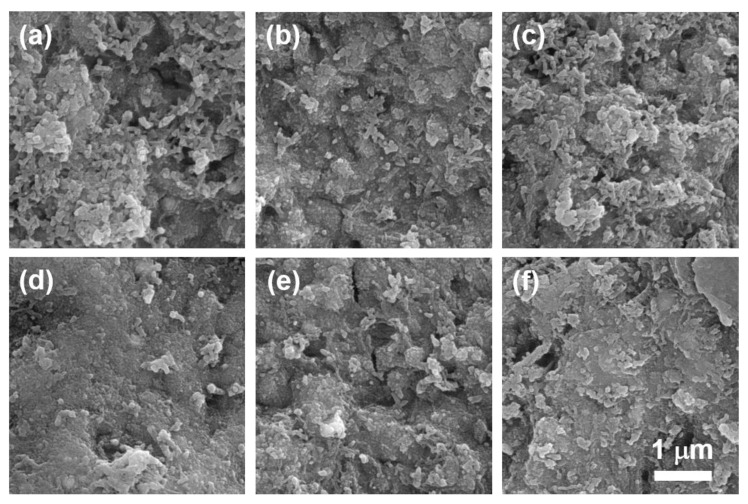
SEM images of the surface morphology of: (**a**) NIP-PANI before sonication; (**b**) NIP-PANI after sonication and before the exposure to PFOS; (**c**) NIP-PANI after the exposure to PFOS; (**d**) PFOS-MIP-PANI before sonication; (**e**) PFOS-MIP-PANI after sonication and before the exposure to PFOS; (**f**) PFOS-MIP-PANI after the exposure to PFOS. The scale bar denotes 1 μm in length.

The surface morphology of PFOS-MIP-PANI and NIP-PANI electrodes are shown in Figure 4. Before sonication treatment of the surface, PFOS-MIP-PANI exhibits a continuous phase with a few clusters and several nanosized spherical crystals of PANI, while NIP-PANI shows irregular clusters as well as spherical crystals of PANI without a relatively continuous microstructure (Figure 4a,d). In Figure 4b,e, after the sonication, NIP-PANI and PFOS-MIP-PANI electrodes do not have a significant difference in surface morphology. The SEM images of the surfaces of PFOS-MIP-PANI and NIP-PANI after the exposure to PFOS are shown in Figure 4c,f, respectively. NIP-PANI shows the morphology similar to the surface before sonication (Figure 4a) and before the exposure to PFOS (Figure 4b), upon which several fragmented and irregular clusters as well as nanosized spherical crystals exist. However, the continuous phase of clusters of a larger size than appear on the surface of NIP-PANI after exposure to PFOS reappear on PFOS-MIP-PANI as shown in Figure 4f. The larger and more continuous domain of clusters and spherical nanocrystalline structures on PFOS-MIP-PANI in Figure 4f are comparable with its surface before sonication where PFOS templates were embedded in the PANI matrix (Figure 4d).

### 3.4. PFOS Detection on PFOS-MIP-PANI Electrodes

**Figure 5:**
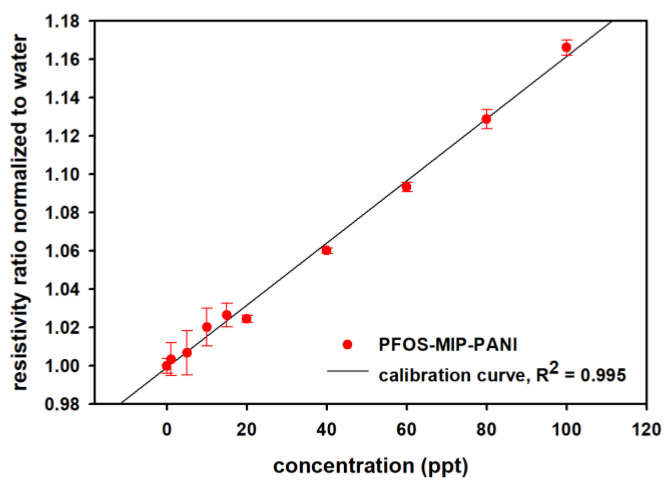
The calibration curve of PFOS-MIP-PANI exposed to various concentrations of PFOS aqueous solutions. The resistivity ratios were normalized to the resistivity ratio of DI water. The data points denote the averages of repeating measurements of DC resistance of at least three devices, from which the standard deviations are also calculated and marked as error bars.

The long-term stability of the PANI paper strips was concluded by measuring the resistivity for 11 weeks as shown in Figure 5. The resistivity of the PANI on the paper substrate in air was stable, suggesting a remarkable stability of the proposed molecularly imprinted paper sensor. PFOS aqueous solutions with concentrations from 0–100 ppt (pH = 7) were prepared for evaluating the detection performance of PFOS-MIP-PANI electrodes. The calibration curve and the linear regression model of resistivity ratios of PFOS-MIP-PANI electrodes as a function of the concentration of PFOS are shown in Figure 5. The calibration curve demonstrates a strong linear relationship between the resistivity ratios and concentrations of PFOS. The linear regression equation of y = 0.0016x + 0.9992 and the coefficient of determination (R^2^) of 0.995 were estimated by the linear regression model calculated by SigmaPlot. The linear range is from 1 to 100 ppt. According to Equations (3) and (4), the limit of detection is estimated to be 1.02 ppt.

### 3.5. Selectivity among Relevant PFCs

**Figure 6:**
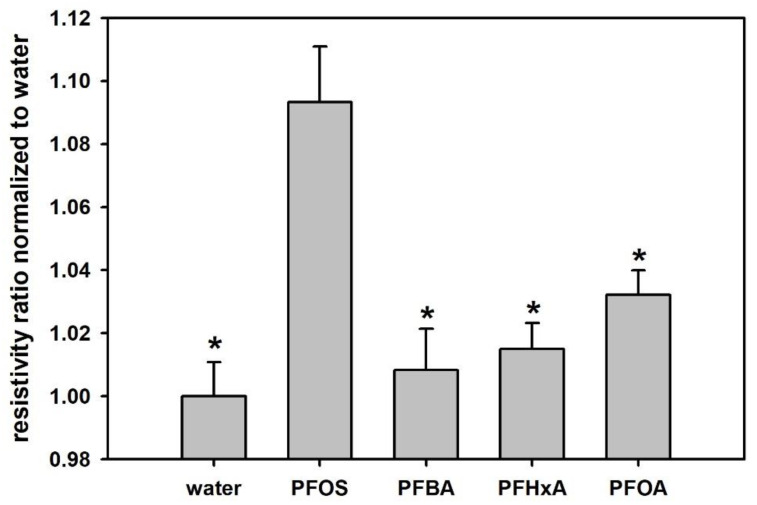
The selectivity of PFOS-MIP-PANI to PFOS, PFBA, PFHxA, and PFOA. The concentration for each chemical in the aqueous samples was 70 ppt. The data were normalized to water resistivity. Each bar denotes the average of DC resistivity ratios measured from three devices, and the standard deviations are also calculated and marked as the error bars. The asterisks represent significance (*p* < 0.05) lower than the response of PFOS.

The selectivity of PFOS-MIP-PANI to PFOS and the other three relevant PFCs of PFBA, PFHxA, and PFOA is shown in Figure 6. After exposure to various PFC samples with a concentration of 70 ppt, PFOS-MIP-PANI demonstrates the largest resistivity ratio to PFOS among other PFC chemical compounds. PFOS-MIP-PANI also shows statistical significance (*p* < 0.05) between PFOS and relevant PFCs, indicating the remarkable selectivity. The average responses of the other three types of PFCs slightly increase as the number of carbon atoms in the tail group increases.

## 4. Discussion

To incorporate as much PFOS as possible in molecular imprinting to generate more recognition sites in the PANI matrix, the concentration of PFOS in the template solution was adjusted to 100 ppm, which is close to the solubility of PFOS in water (~550 ppm). In general, concentrations of template would be larger than concentrations of samples to ensure the binding effectiveness. In addition, using paper and conductive polymers as the substrate and the electrodes can achieve high-throughput fabrication and dramatically reduce the cost compared to conventional assays. As a result, for potential forms of utilization, this paper sensor is most likely to be fabricated as a fast-screening or single-use testing kit.

According the ATR-FTIR spectra in Figure 2, the characteristic peak around 1570 cm^−1^ represents C=N stretching of the quinoid form of aromatic rings in the PANI matrix [63]. The aromatic rings have resonance structures as quinoid and benzoic forms while electric charges are transferred through the PANI backbones. Lal et al. found that the quinoid rings of PANI were attributed to the intermolecular interaction between multiwall carbon nanotubes (MWCNT) and the PANI matrix, further improving the thermal stability as the glass transition temperature of the MWCNT-PANI nanocomposite increased [64]. Therefore, this red-shift indicates the potential intermolecular interaction and hybridization between PFOS and PFOS-MIP-PANI, and that possible binding sites could be located on quinoid rings in the PANI matrix. In addition, the characteristic peak at 1375 cm^−1^ of PFOS-MIP-PANI represents the axial stretch of CF_2_ bonding [65], which suggests the presence of PFOS on MIP-PANI electrodes whereas NIP-PANI does not show a similar functional group. The peaks around 1035 cm^−1^ may be attributed to the symmetric C-C stretch of PFOS [65], suggesting additional evidence of PFOS molecules captured on the recognition sites created by molecular imprinting.

In the molecular imprinting process, templates coexist with monomers in the solution during polymerization, after which templates must be removed and leave cavities as recognition sites to target molecules. Therefore, the procedure of removing template molecules from a polymer matrix plays an important role for synthesizing MIPs. Sonication in a cosolvent of acetic acid/methanol was utilized to extract PFOS templates from the PANI matrix. Herein, the XPS experiments were to investigate the efficiency of PFOS template removal from the PANI substrate. Before sonication, PFOS-MIP-PANI contained PFOS templates and its F1s spectrum shows a peak around 688 eV which represents aliphatic fluorine (CF_2_) bonding [66]. After sonication in the cosolvent, the corresponding CF_2_ peak in the F1s spectrum disappears, suggesting the successful removal of PFOS templates from PFOS-MIP-PANI. Once the PFOS-MIP-PANI was exposed to PFOS in the detection, PFOS molecules were captured by the molecularly imprinted recognition sites in PFOS-MIP-PANI proven by the reemerged CF_2_ peak in the XPS results. In contrast, NIP-PANI did not show the aliphatic fluorine peaks due to the lack of specific binding cavities.

The similar red-shift behavior related to nitrogen bonding in the ATR-FTIR spectra is also found in the N1s XPS spectra (Figure 3b), indicating the intermolecular interaction between PFOS and nitrogen atoms in PANI. Depending on the resonance structures, PANI could have N1s XPS peaks combined with binding energies ranging from 398.5 eV (imine, C=N-C) to 399.5 eV (amine, C-NH-C) and 401.5 eV (protonated imine, C=(NH^+^)-C) [67]. The red-shift towards 398.5 eV implies that PFOS-MIP-PANI shared more characteristics of imine functional groups than NIP-PANI did, and the tendency of imine structures agrees with the quinoid rings referred to the ATR-FTIR spectra. As a result, quinoid rings and nitrogen atoms could be the functional groups of the recognition sites that allow PFOS-MIP-PANI to detect PFOS.

Before sonication, the presence of PFOS during polymerization may result in the structural refinement of irregular and fragmented clusters, leading to a continuous phase embedding the PFOS templates in the PFOS-MIP-PANI. The removal process disposed of the PFOS templates and brought the surface morphology back to fragmented clusters. Finally, after dispensing the sample solutions on the surface, the PFOS-MIP-PANI recognized and captured PFOS molecules as if they were embraced after polymerization, causing a continuous phase to emerge again. On the contrary, NIP-PANI did not have a significant transformation of the surface morphology between those steps. These results provide a morphological point of view to describe the behavior of detaching templates and recognizing targets for MIPs.

The long-term stability study was to validate the synthesis procedures and the stability of the synthetic PANI platform we used. The remarkable durability of the polyaniline synthesized on the polyester paper strips may be attributed to the hydrophobic surface and a great affinity with aniline monomers that result in a dense and continuous film on the paper substrate. The relatively hydrophobic paper also allows the strips to remain whole in the solution without being disintegrated by vigorous stirring. There are small deviations found in Figure S1, which may due to changes of temperature and humidity that is less than 10% of resistivity. The reaction time of 30 min after dispensing PFOS samples was used to ensure that the detection reached an equilibrium. The calibration curve shows a positive and linear correlation of normalized resistivity ratios as a function of PFOS concentrations in aqueous solutions, which means that the resistance of PFOS-MIP-PANI electrodes increased after exposure to PFOS. The larger standard deviation of the responses at the lower concentrations of PFOS may result from the small variation in resistance. The extremely low concentration of PFOS may also limit the association between PFOS and PFOS-MIP-PANI and prolong the time for reaching equilibrium, increasing the standard deviation.

Molecular imprinting technology provides a promising method for fabricating specific binding sites, which benefit from the intermolecular interaction between templates and polymer matrix. Since the approach of detection depends on the intermolecular interaction and structural complementarity, we used relevant PFCs with a different functional group (carboxylic acid) and different lengths of aliphatic groups from four to eight carbons (PFBA, PFHxA, and PFOA) to evaluate the effectiveness of molecular imprinting for PFOS detection. The slightly increased resistivity ratios for the longer PFCs might result from the more similar geometry to PFOS. Particularly, PFOA also consists of eight carbons and a similar functional group of carboxylic acid, making PFOA be more responsive to PFOS-MIP-PANI than PFBA and PFHxA are. However, the responses for those shorter PFCs or PFOA still have statistical significance to the response of PFOS as shown in Figure 6. Molecular imprinting of PFOS on PANI can still fabricate receptor-like structures facilitating the recognition of target molecules; therefore, PFOS-MIP-PANI demonstrates excellent selectivity to PFOS among other comparable PFCs.

**Figure 7:**
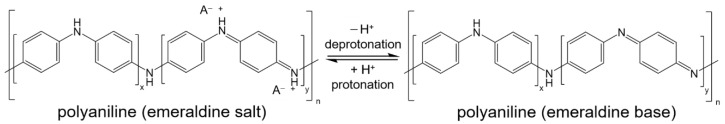
The transformation between the forms of polyaniline emeraldine salt and emeraldine base. A^−^ denotes the counter ion.

The detection mechanism of molecular-imprinted PANI has been proposed from the obstruction of electric hole transfer caused by the occupation of target molecules in the recognition sites on the surface where electric holes transfer through [47]. The absorption of target molecules can modulate the conductivity of PANI. As shown in Figure 7, polyaniline exists in two types of configurations: emeraldine salt and emeraldine base. Due to presence of the positive charges as the charge carriers for the electrical conductance through the PANI matrix which is like a p-type semiconductor, emeraldine salt is conductive while emeraldine base is much less so. In the synthesis of PANI, the addition of HCl as the dopant provides the conductance of the PANI by the emeraldine salt. PFOS is an aliphatic acid that is rich in negative charges and electron long pairs, which could attract the electron holes on nitrogen atoms of PANI and bring the functional groups from the bulk to the surface during the molecular imprinting polymerization, creating a mutually compensated structure of PFOS and PANI matrix by means of the electrostatic interaction. When PFOS samples are dispensed on the surface of the PFOS-MIP-PANI electrodes, where the recognition sites are rich in positive charges, the negative charges of the PFOS might bind with the PANI by neutralizing those electron holes, resulting in a reduction of the number of charge carriers as well as in the conductivity. This also agrees with the increased characteristics of imine groups indicated by the XPS spectra in Figure 3, suggesting that the partial emeraldine salt in the PANI might have been transferred into the emeraldine base which is less conductive, causing the increased resistance of the calibration curve in Figure 5. Since the pH value of the PFOS sample solution was close to 7, the exposure of trace PFOS might not be able to reveal a significant colorimetric change of the PANI electrode from green (emeraldine salt) to blue (emeraldine base). Instead, the absorption of PFOS is still demonstrated in the FTIR and XPS spectra, and the peak-shift in XPS indicates more characteristics of imine groups that contribute to the less-conductive emeraldine base. Although XPS is not designed for quantitative analysis due to lack of standards, and the actual balance of salt form and base form implied by the addition of PFOS is yet to be determined, the spectra still provide a comparable and corresponsive proof of PFOS detection and a loss of conductivity.

Challenges of the paper-based sensor include the selectivity and sensitivity that limit the capability of distinguishing various types of PFCs at extremely low concentrations. Additionally, the stability of the electrical responses of resistance could be improved. A well-defined and consistent fabrication process could further enhance the detection efficiency and accuracy and reduce the cost of the entire device. In the future, development of the PFOS-MIP-PANI paper sensor will focus on improving the selectivity as well as the sensitivity and optimizing the fabrication process, which could provide more high-throughput and reliable signals.

## 5. Conclusions

In this study, we have designed a molecularly imprinted and low-cost PANI paper-based sensor for detecting PFOS in aqueous solution that has potential of replacing conventional assays that are expensive and complicated. The PFOS-MIP-PANI paper sensor demonstrated a linear calibration curve of resistivity as a function of PFOS concentrations. It was proposed that the detection mechanism was associated with quinoid rings and nitrogen atoms in the PANI matrix where intermolecular interactions between PFOS-MIP-PANI and PFOS are deployed, which is suggested by the XPS and ATR-FTIR spectra. The red-shift transformation in the spectra may indicate more characteristics of the non-conductive configuration of PANI, which agrees with the resistivity change of the PFOS-MIP-PANI strips upon exposure to PFOS. The simple fabrication, the long-term durability, and the accurate detection of the molecularly imprinted paper-based sensor provides the potential for low-cost sensing and large-scale monitoring of PFOS in wastewater and thus addressing relevant public health issues, particularly in underserved areas.

## Supplementary Materials

The following are available online at https://www.mdpi.com/1424-8220/20/24/7301/s1: Figure S1: Results of the long-term stability of PANI electrodes on paper (*n* = 4).
