# Supplementary material for: Perfluorooctanesulfonic Acid Detection Using Molecularly Imprinted Polyaniline on a Paper Substrate
Source: Sensors (Basel). 2020 Dec 19;20(24):7301. doi: 10.3390/s20247301 (PMC7765859; doi:10.3390/s20247301)
Supplement: Supplementary file 1 [file sensors-20-07301-s001.pdf]

sensors

MDPI

Supplementary Materials:

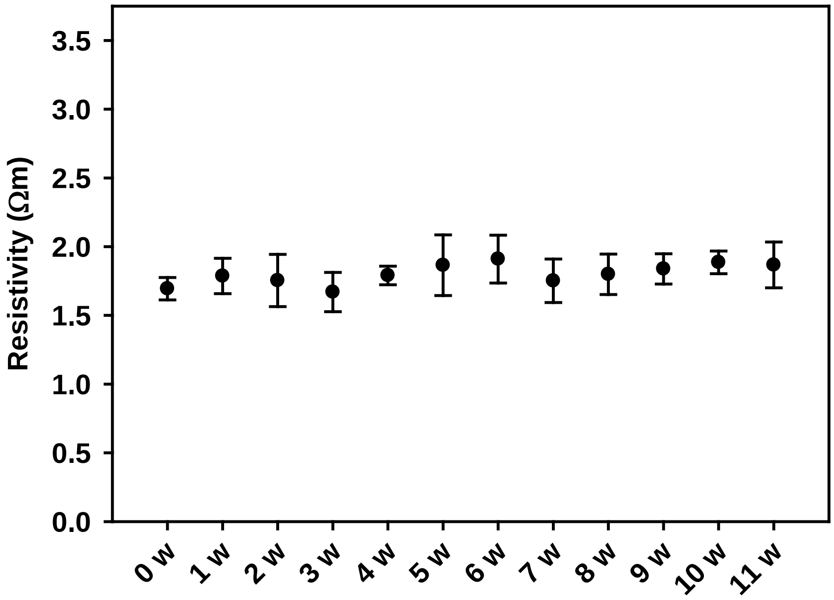

Figure S1. Results of the long-term stability of PANI electrodes on paper. (n = 4).
